# The many antibiotic resistance and tolerance strategies of *Pseudomonasaeruginosa*

**DOI:** 10.1016/j.bioflm.2021.100056

**Published:** 2021-08-21

**Authors:** Devin Sindeldecker, Paul Stoodley

**Affiliations:** a Department of Microbial Infection and Immunity, The Ohio State University, Columbus, OH, USA; b Biomedical Sciences Graduate Program, The Ohio State University, Columbus, OH, USA; c Department of Orthopaedics, The Ohio State University, Columbus, OH, USA; d National Center for Advanced Tribology at Southampton (nCATS), Mechanical Engineering, University of Southampton, Southampton, UK

**Keywords:** Pseudomonas, Antibiotic tolerance, Antibiotic resistance, Biofilm

## Abstract

*Pseudomonas aeruginosa* is a bacterial pathogen associated with a wide range of infections and utilizes several strategies to establish and maintain infection including biofilm production, multidrug resistance, and antibiotic tolerance. Multidrug resistance in *P. aeruginosa*, as well as in all other bacterial pathogens, is a growing concern. Aminoglycoside resistance, in particular, is a major concern in *P. aeruginosa* infections and must be better understood in order to maintain effective clinical treatment. In this review, the various antibiotic resistance and tolerance mechanisms of *Pseudomonas* are explored including: classic mutation driven resistance, adaptive resistance, persister cells, small colony variants, phoenix colonies, and biofilms. It is important to further characterize each of these phenotypes and continue to evaluate antibiotic surviving isolates for novel driving mechanisms, so that we are better prepared to combat the rising number of recurrent and recalcitrant infections.

## 1 Introduction

*Pseudomonas aeruginosa* is a Gram-negative, opportunistic, bacterial pathogen associated with a wide range of infections including cystic fibrosis (CF) associated lung disease, post-surgical infections, and chronic wound infections [[1], [2], [3], [4]]. *P. aeruginosa* has several strategies which it uses to establish and maintain infection including biofilm production, multidrug resistance, and antibiotic tolerance [[5], [6], [7], [8]]. Along with several other bacterial pathogens, multidrug resistance in *P. aeruginosa* is a growing concern [[9], [10], [11], [12], [13]]. In addition, *P. aeruginosa* CF isolates have been shown to be hypermutable, further raising the concern for antimicrobial tolerance and resistance to develop [14]. Aminoglycoside resistance, in particular, is a growing concern in *P. aeruginosa* [[15], [16], [17]] and is something which must be understood and accounted for in clinical treatment plans.

*P. aeruginosa* has multiple antibiotic resistance and tolerance phenotypes which could allow survival of a bacterial population during antibiotic treatment of an infection. These phenotypes are highly diverse in not only their mechanisms of development but also in the extent to which they are able to survive in the presence of antibiotics. Antibiotic tolerance has also been found to allow for development of complete antibiotic resistance [18], further showing the importance of understanding how these phenotypes develop and function in order to prevent recurrent and recalcitrant infections. In this review, various resistance and tolerance phenotypes will be summarized in terms of their mechanisms of development and survival despite the presence of antibiotics.

## 2 Antibiotic resistance

Antibiotic resistance is characterized primarily by genetic alterations which allow cells to actively resist killing by antibiotics. This can be accomplished through antibiotic target site modification, enzymatic degradation of the antibiotic, or an increase in expression of efflux pump genes [[19], [20], [21]]. Additionally, there are other mechanisms which confer antibiotic resistance, including heteroresistance and adaptive resistance.

### 2.1 Mutation driven antibiotic resistance

“Classical” antibiotic resistance is driven by either stable mutations or horizontal gene transfer of plasmids harboring resistance genes, both of which allow bacteria to survive in the presence of antibiotics at both high concentrations and over repeated exposures. Treatment of *P. aeruginosa* is primarily accomplished using aminoglycosides such as gentamicin or tobramycin. Aminoglycoside resistance through modifying enzymes which inactivate the aminoglycoside has been known to exist since the 1960's and 1970's [[21], [22], [23]]. These enzymes often phosphorylate or adenylate the antibiotics and multiple modifying enzymes are often harbored in a single genome, allowing for broad-spectrum antibiotic disruption [[24], [25], [26]]. In addition, modification of membrane permeability can lead to a decrease in the uptake of antibiotics [27,28], and, additionally, the presence of efflux pumps such as the MexXY pump (in aminoglycosides) or the MexCD-OprJ and MexEF-OprN pumps (in fluoroquinolones) serve to further prevent antibiotics from accumulating intracellularly [24,[29], [30], [31], [32]]. Target site modification (ribosomal in the case of aminoglycosides, or DNA gyrase in fluoroquinolones) has also been noted, leading to a lack of binding of the antibiotic to its target [27,33]. Antibiotic resistance can also be conferred through horizontal gene transfer, in which antibiotic modifying enzyme genes can be acquired by plasmid transfer from other species of bacteria [34,35]. Clinically, antibiotic resistance is a growing concern. A recent study on 60 P. aeruginosa strains isolated from burn patients found that 90% were resistant to at least one antibiotic and 94% of the isolates were multidrug resistant [25]. Another study on *P. aeruginosa* clinical isolates found overexpression of MexXY-OprM in 53% of strains, indicating the importance of efflux pumps as well in a clinical setting [24].

### 2.2 Heteroresistance

In additional to classical antibiotic resistance, in which a complete population exhibits the phenotype, heteroresistance is a classification characterized by a small subset of genetically resistant bacteria hiding within a population which is overall susceptible to the antibiotic [36,37]. During antibiotic exposure, the majority of the population is killed leaving the resistant subset behind to recolonize as an antibiotic recalcitrant infection [38,39]. Although this is similar to the early stages of classical resistance development, it is important to note that heteroresistance is unstable and can revert to an antibiotic susceptible population where the antibiotic pressure is removed [36]. This instability, combined with the low frequency of resistant cells within the population, leads to difficulties in detection of heteroresistance [36]. Clinically, antibiograms are charts used to determine the susceptibility of a culture to various antibiotics. The most commonly used methods to generate an antibiogram are by disc diffusion or Etest assays. Unfortunately, heteroresistance is difficult to identify using traditional antibiogram methods due to the possibility of the overall population appearing susceptible during the initial assay if the resistant population is too small to be detected [40,41] and it is possible that this could lead to treatment failure [41,42]. Population assay profiling (PAP) uses a dilution series of antibiotic concentrations to allow the heteroresistant population to emerge and be visualized [43]. Heteroresistance has also been linked to spontaneous, unstable tandem amplifications of known resistance genes across different bacterial species and in response to various antibiotics [44]. In order to combat the presence of these resistance mechanisms in a heteroresistant population, Band et al. propose using combination antibiotic therapy to exploit these populations in a clinical setting [45]. Combination antibiotic therapy would be effective in treating a population containing multiple heteroresistant subpopulations by targeting multiple subcellular sites. This would overcome the resistance mechanism of each subpopulation and allow for complete killing of all of the bacteria regardless of the presence of heteroresistance.

### 2.3 Adaptive resistance

Another antibiotic survival mechanism of *P. aeruginosa*, termed adaptive resistance, is characterized by a transient resistance to antibiotics. Adaptive resistance in *P. aeruginosa* was first identified clinically in sputum samples from CF patients in 1996 [46]. While the molecular mechanisms of resistance are not fully understood, this phenotype is primarily driven by environmental stimuli such as antimicrobial exposure, pH changes, anaerobic environments, and starvation. Adaptive resistance has also been highly linked to swarming motility, biofilm development, and a transient upregulation of the MexXY-OprM efflux pump [[47], [48], [49]]. Once the antibiotic pressure is removed, the adaptive resistance bacteria are able to revert to a wild-type level of antibiotic susceptibility [48]. Currently, the presence of an adaptive resistance phenotype being present in a clinical setting is speculative, and further research is needed to explore the danger which this phenotype may present.

## 3 Antibiotic tolerance

In addition to antibiotic resistance, antibiotic tolerance is an area of increased concern, especially given the potential of tolerance leading to population resistance over time [18]. Antibiotic tolerance is generally differentiated from antibiotic resistance by a lack of a stable phenotype. Tolerance is characterized as an ability to survive transient exposure to high concentrations of antibiotic without a change in the minimum inhibitory concentration (MIC) for the organism. This is often achieved by altering essential bacterial processes [50].

### 3.1 Persister cells

**Fig. 1:**
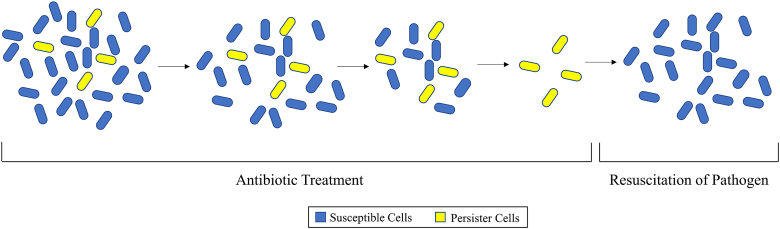
**–** Progression of persister cells during antibiotic treatment. During initial antibiotic exposure, the population contains a subset of persister cells which remain in a dormant state. As time progresses, the antibiotic is able to kill susceptible cells, while the dormant persister cells survive. After removal of the antibiotic, the susceptible cells are dead, yet the persister cells can resuscitate and regenerate the susceptible population. Adapted from Renbarger et al. [52].

Persister cells are an antibiotic tolerant phenotype of bacteria which enter a metabolically inactive state of dormancy but return to a wild-type level of antibiotic susceptibility once antibiotic concentrations drop below the MIC leading to a population which is again susceptible to the antibiotic (Fig. 1) [5,51,52]. They were first described in *Staphylococcus aureus* by Hobby et al., in 1942 [53]. Two years later, Joseph Bigger further described the phenotype in *Staphylococcus pyogenes*, adding that while persister cells were able to survive antibiotics (penicillin), they were not genetically different than wild-type [54]. Further studies have implicated toxin-antitoxin (TA) systems in the mechanism behind persister cell tolerance [5,[55], [56], [57]]. TA systems are comprised of a stable, protein toxin which disrupts essential cellular processes as well as an antitoxin which prevents toxicity [57]. Overproduction of the toxin portion of a TA system relative to antitoxin production leads to an autotoxicity induced dormancy state. Two TA systems have been identified in *Escherichia coli* which led to the development of the persister cell phenotype, the MqsR/MqsA system and the TisB/IstR-1 system [58,59]. Within the MqsR/MqsA system, MqsR leads to diminished translation and ability to respond to cellular stresses leading to a state of dormancy [[60], [61], [62], [63]]. For the TisB/IstR-1 system, the TisB toxin decreases both the proton motive force and ATP leading to cellular dormancy [22]. Although *E. coli* persister cells have been studied extensively, these TA systems do not have homologs in *P. aeruginosa* and little is known about the mechanisms behind *P. aeruginosa* persister cell development despite a high level of emergence specifically within CF patients [64]. Persister cells develop at a low rate (~1% of the population [65]), however, it is a major concern due to the possibility of them leading to recurrent infections [8], although this has yet to be confirmed in a clinical study.

### 3.2 Small colony variants

Small colony variants (SCV) are phenotypic variants directly associated with antibiotic tolerance and persistent infections. In *P. aeruginosa*, they were first described in CF associated *P. aeruginosa* respiratory tract infections [66]. After their discovery, the first clinical investigation was performed from 1996 to 1998 [67]. After testing sputum from 86 CF patients for *P. aeruginosa* SCVs, it was found that 33 patient samples contained isolates from this phenotype. SCVs are typically characterized by their small size relative to wild-type. This small colony size is due to either a slower growth rate like that exhibited by *Staphylococcus aureus* SCVs [68,69], or more commonly by extracellular matrix overproduction in *Pseudomonas*' rugose SCVs (RSCVs), which also allow SCVs to be tolerant to a range of antibiotic classes [67,69]. SCVs can be differentiated from RSCVs by their appearance. SCVs are typically small and smooth colonies, whereas RSCVs, also known as wrinkly spreader colonies, have a rough appearance due to the overproduction of matrix components [69,70]. Drenkard and Ausubel showed that *P. aeruginosa* RSCVs could be induced by the addition of kanamycin to culturing media and were able to link the phenotype to the cyclic-di-GMP (cdG) phosphodiesterase gene, pvrR [71]. Additionally, D'Argenio et al. identified another gene implicated in RSCV formation within the lab strain *P. aeruginosa* PAO1, the WspR diguanylate cyclase (DGC) [72]. cdG is produced when DGC joins two molecules of GTP [73]. cdG is highly promiscuous and binds to transcriptional regulators [[74], [75], [76]]. Within RSCVs, intracellular levels of cdG have been found to be elevated, leading to transcriptional changes including the overproduction of exopolysaccharides, fimbrial adhesins, Psl, Pel, and alginate [[77], [78], [79], [80], [81], [82]]. These changes contribute to both the rough, morphological presentation of RSCVs as well as the antibiotic tolerance phenotype which allows RSCVs to survive therapeutic interventions. Antibiotic tolerance of RSCVs is likely due to the hyperbiofilm state produced by overproduction of the extracellular matrix components. Clinically, RSCVs have been seen for decades and continue to be a concerning issue, particularly within the field of cystic fibrosis. Additionally, RSCVs have been associated with prolonged antibiotic treatment and poor clinical outcomes [67].

### 3.3 Metabolic alterations

In addition to the aforementioned, well described, antibiotic tolerant phenotypes, other metabolic variants have been identified which are tolerant to antibiotics. In 2019, Schiessl et al. described an antibiotic tolerant phenotype of *P. aeruginosa* which is driven by an alternative metabolism induced in anaerobic or microaerobic environments [83]. This paper proposed that the alternate metabolism uses phenazines which are produced by the *Pseudomonas* cells as an alternative electron acceptor due to the lack of available oxygen. Further research has shown that when glucose and pyruvate are converted into acetate by fermentation, phenazines are able to regenerate the oxidant NAD(P)H by acting as an extracellular electron shuttle and alleviating the redox constraints on the metabolic pathway [84]. While the link is not fully understood, this metabolic phenotype confers tolerance to ciprofloxacin, allowing for survival until oxygen is present again [83]. An overproduction of agmatine has also been associated with antibiotic tolerance in *P. aeruginosa*. Agmatine is a pre-poly-amine intermediate metabolite of the arginine decarboxylase pathway. After observing a correlation between agmatine concentration and CF disease severity, McCurtain et al. further explored the effects of this metabolite on antibiotic tolerance and virulence. It was found that cells harboring an increased amount of agmatine were tolerant to positively charged aminoglycosides and polymyxins but were still susceptible to antibiotics with a neutral charge. It is believed that this is due to membrane stabilization since agmatine is also positively charged [85]. Further studies are needed to better characterize these and other metabolic variants of *P. aeruginosa* including the mechanisms conferring this type of antibiotic tolerance, particularly their role in a clinical setting.

### 3.4 Phoenix colonies

In 2020, Sindeldecker et al. described a novel antibiotic tolerant phenotype which they have termed phoenix colonies [86]. Phoenix colonies are able to grow and remain metabolically active in the presence of antibiotics, even when the antibiotic concentration is > 10 times the MIC. However, after being removed from the antibiotic environment from which they emerged, the phoenix colonies return to a wild-type level of antibiotic susceptibility [86]. The molecular mechanisms behind this phenotype are currently unknown and much work is needed to better characterize and understand their antibiotic survival and its implications. Similar to heteroresistance, phoenix colonies appear to have avoided detection until now due to the limitations of conventional assays. Anecdotally, due to relatively short incubations times (~24 h), colonies do not typically arise within the zone of inhibition or zone of clearance of a bacterial population. Those which do arise have been considered to be resistant mutants. The methods used to detect phoenix colonies, involved incubating the bacteria for an extended period of time (120 h) before replica plating onto both media containing and lacking antibiotic in order to differentiate between resistant colonies and any tolerance mechanisms which may be present [86]. Additionally, the PAP assay for heteroresistance uses cultures equivalent to a 0.5 McFarland standard, which are approximately 1 × 10^8^ CFU/mL [87]. The higher concentrations of bacteria (~5 × 10^9^ CFU/mL) used to detect phoenix colonies provide a more sensitive system which may be able to further detect heteroresistance [86]. As phoenix colonies have only recently been discovered, it has yet to be confirmed whether or not they may exist in a clinical setting. It is also important to note that the field of antibiotic tolerant phenotypes is still advancing, leading to new tolerant phenotypes continuing to be discovered.

### 3.5 Biofilm populations

**Fig. 2:**
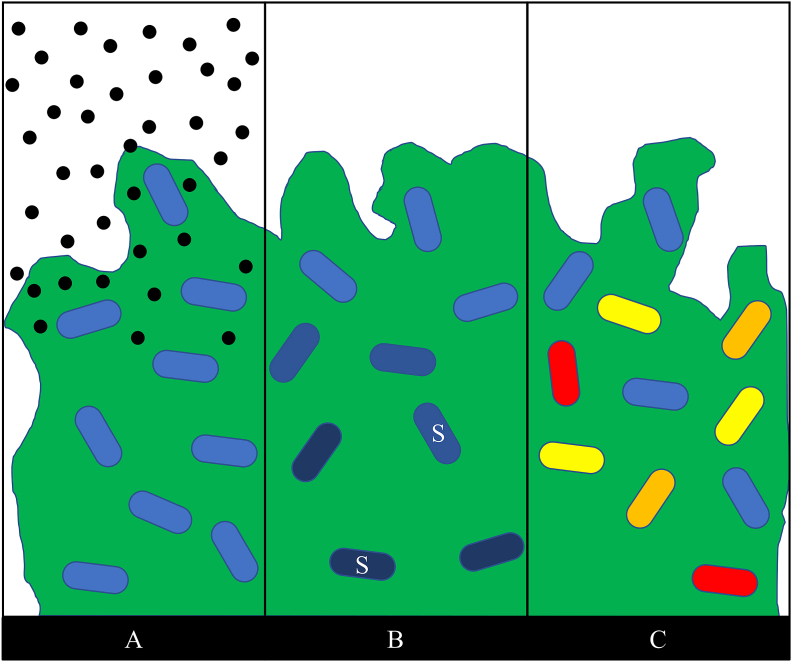
Four mechanisms of antibiotic survival in *P. aeruginosa* biofilms. A – Antibiotic (black circles) penetration is restricted, preventing complete killing of biofilm cells; B – a concentration gradient of oxygen and nutrients leads to regions of slow or non-growing bacteria (shaded cells) deeper within the biofilm, some cells within the biofilm may also exhibit an increase in the SOS response (white “S”) due to nutrient depletion; C – the large population of cells in the biofilm increase the chances for persister cells (yellow), phoenix colonies (red), or resistant mutants (orange) to emerge. Adapted from P. S. Stewart [106]. (For interpretation of the references to colour in this figure legend, the reader is referred to the Web version of this article.)

In addition to phenotypes which occur in single cells of a population, antibiotic tolerance can also be conferred at the population level though mechanisms such as biofilm formation. Biofilms are populations of bacteria which conglomerate and encase themselves in an extracellular polymeric substance (EPS) [88]. The EPS matrix is comprised of polysaccharides, proteins, eDNA, and lipids and provides a scaffolding structure for the bacteria within the biofilm [89,90]. In *P. aeruginosa* specifically, the main components of the EPS are Pel, Psl, and alginate, three exopolysaccharides [[89], [90], [91], [92]]. cdG is an important transcriptional regulator for the biofilm phenotype and causes an increase in production of adhesins and EPS components [[93], [94], [95]]. Quorum sensing is also an important function for control of biofilm formation [96] and consists of two major systems, Las and Rhl [97]. One important characteristic of biofilms is their ability to survive high concentrations of antibiotics. This antibiotic tolerance is conferred through a number of mechanisms [4], the most basic of which is a restriction in antibiotic penetration into the biofilm (Fig. 2a). This restriction primarily effects charged antibiotics as they are bound up by other charged components of the EPS [98,99]. This antibiotic binding protects bacteria which are deeper within the biofilm, as the antibiotics are hindered from reaching them. In addition to antibiotics being unable to effectively penetrate the biofilm, nutrients and oxygen are also limited deep within the biofilm leading to a slower growth phenotype (Fig. 2b). Nutrient depletion also leads to an increase in the SOS and stringent responses (Fig. 2b) which have also been shown to play a role in tolerance [4,100,101]. Additionally, the large population increases the chance for the emergence of persister cells, phoenix colonies, resistant mutants, and any other small population phenotype (Fig. 2c). Both the slow growth and persister cell phenotypes exhibit an increased tolerance to antibiotics [55,102]. As mentioned previously, the survival of persister cells could possibly lead to a recurrent infection [8]. Clinically, biofilm related P. aeruginosa infections are commonly observed in chronic obstructive pulmonary disorder, cystic fibrosis, urinary tract infections, catheterization, intubation, and surgical site infections [2,[103], [104], [105]]. Biofilm related infections are considered especially serious due to the difficult in achieving complete killing and clearance of the biofilm.

## 4 Conclusions

**Fig. 3:**
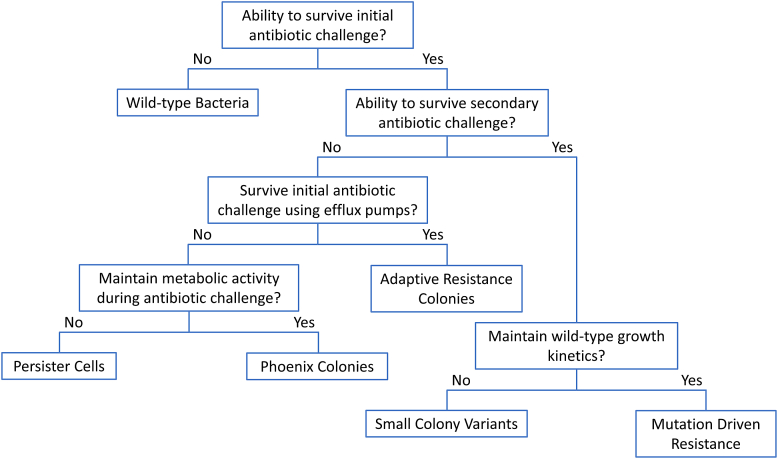
Antibiotic Tolerant and Resistant Phenotype Comparisons. Flow chart comparing the differences between the most common antibiotic tolerant and resistant phenotypes in *P. aeruginosa*. The range of phenotypes span fully susceptible wild-type bacteria, transiently tolerant phenotypes, and fully resistant bacteria driven by genetic mutations.

Numerous antibiotic tolerant and resistant phenotypes exist in *P. aeruginosa*, at both the single cell (Fig. 3) and population levels. Both antibiotic tolerance and antibiotic resistance are growing issues throughout many pathogenic species, including *P. aeruginosa*. Clinically, classical antibiotic resistance, heteroresistance, RSCVs, and biofilms have been implicated in *P. aeruginosa* infections [25,41,67,[107], [108], [109]]. The presence of these antibiotic resistance and antibiotic tolerance phenotypes is extremely concerning not only due to the difficulty in treating infections of this nature but also due to the increased severity of these infections [67,89]. As antibiotic resistance and tolerance continues to emerge, the morbidity and mortality associated with these infections will also likely increase. An understanding of the mechanisms by which *P. aeruginosa* is able to survive antibiotic therapeutics is fundamental in not only the clinical setting but also in the laboratory setting, as it is important to be able to differentiate between the various phenotypes when performing any research related to antibiotic therapies. It is also important to further characterize these phenotypes and to continue to evaluate antibiotic surviving isolates for novel driving mechanisms, so that we may be able to further our knowledge and combat the rising number of reoccurring, persisting, and recalcitrant infections.

## Declaration of competing interest

The authors declare the following financial interests/personal relationships which may be considered as potential competing interests: Paul Stoodley reports financial support was provided by National Institutes of Health (R01 NIH-GM124436).
